# Multiplex single-cell visualization of nucleic acids and protein during HIV infection

**DOI:** 10.1038/s41467-017-01693-z

**Published:** 2017-12-01

**Authors:** Maritza Puray-Chavez, Philip R. Tedbury, Andrew D. Huber, Obiaara B. Ukah, Vincent Yapo, Dandan Liu, Juan Ji, Jennifer J. Wolf, Alan N. Engelman, Stefan G. Sarafianos

**Affiliations:** 1 CS Bond Life Sciences Center, University of Missouri, Columbia, MO 65211 USA; 2 Department of Molecular Microbiology & Immunology, University of Missouri School of Medicine, Columbia, MO 65212 USA; 3 Department of Veterinary Pathobiology, University of Missouri, Columbia, MO 65211 USA; 4 Department of Cancer Immunology and Virology, Dana-Farber Cancer Institute, Boston, MA 02215 USA; 5 Department of Medicine, Harvard Medical School, Boston, MA 02115 USA; 6 Department of Biochemistry, University of Missouri, Columbia, MO 65201 USA; 7 Laboratory of Biochemical Pharmacology, Department of Pediatrics, Emory University School of Medicine, Atlanta, GA 30332 USA

## Abstract

Technical limitations in simultaneous microscopic visualization of RNA, DNA, and proteins of HIV have curtailed progress in this field. To address this need we develop a microscopy approach, multiplex immunofluorescent cell-based detection of DNA, RNA and Protein (MICDDRP), which is based on branched DNA in situ hybridization technology. MICDDRP enables simultaneous single-cell visualization of HIV (a) spliced and unspliced RNA, (b) cytoplasmic and nuclear DNA, and (c) Gag. We use MICDDRP to visualize incoming capsid cores containing RNA and/or nascent DNA and follow reverse transcription kinetics. We also report transcriptional “bursts” of nascent RNA from integrated proviral DNA, and concomitant HIV-1, HIV-2 transcription in co-infected cells. MICDDRP can be used to simultaneously detect multiple viral nucleic acid intermediates, characterize the effects of host factors or drugs on steps of the HIV life cycle, or its reactivation from the latent state, thus facilitating the development of antivirals and latency reactivating agents.

## Abstract (web summary)

Technical limitations in simultaneous microscopic visualization of HIV transcription from individual integration sites have curtailed progress in the field. Here the authors report a branched DNA in situ hybridization method for direct single-cell visualization of HIV DNA, RNA, and protein.

## Introduction

Despite progress in nucleic acid visualization techniques, visualization of HIV transcription from individual integration sites has proven elusive. Moreover, there is a need for an integrated approach to simultaneously monitor changes in spliced and unspliced viral RNA (vRNA), viral DNA (vDNA), and proteins at a single-cell level, during the various steps of the HIV replication cycle.

Various approaches have been reported over the past few years, for the combined imaging of HIV nucleic acids and proteins. One of the first approaches to allow visualization of integrated HIV-1 proviruses exploited the recruitment of specific histones to sites of DNA damage, in combination with a reporter virus containing a rare restriction site^1^. This single-cell imaging of HIV-1 provirus (SCIP) approach provided sensitive labeling of integrated provirus, but not unintegrated vDNA, in apparent contrast to later techniques. Others exploited 5-ethynyl-2-deoxyuridine (EdU), which can be incorporated into nascent DNA and then labeled with fluorescent azides by click chemistry^2, 3^. This approach can be used with native virus, rather than a reporter virus, and has been successfully employed in non-dividing cells. The use of EdU is challenging in dividing cells; however, as EdU is incorporated into the genome of the infected cell, generating high background. For nucleic acid labeling in dividing cells, several groups have applied variations of fluorescence in situ hybridization (FISH); either immuno-DNA FISH^4^ or branched DNA (bDNA)-FISH^5^. These FISH approaches allowed investigators to examine the vDNA localization at various points during infection, and to identify the number and position of viral integration sites in the host genome. Each method brings strengths and shortcomings, such as being limited to either RNA or DNA labeling, or requiring treatment of the infected cell during reverse transcription to label the viral genome.

Here we describe multiplex immunofluorescent cell-based detection of DNA, RNA and protein (MICDDRP), a bDNA-FISH method with the ability to label the native nucleic acids of the HIV-1 replication cycle, and show how it can be used to track various intermediates of HIV replication, focusing on the kinetics with which various species appear following infection. We follow the appearance of vDNA, nuclear import of vDNA, vRNA transcription from integrated vDNA, splicing of vRNA and nuclear export of vRNA. The ability to visualize these nucleic acid intermediates in the context of viral or host proteins will advance efforts to elucidate mechanisms of antiviral inhibition by small molecules or host restriction factors, enhance our understanding of latency reactivation, and further efforts for novel drug development.

## Results

### Specific visualization of HIV-1 RNA and DNA

FISH techniques have been established for detection of nucleic acids in cells, but lack the sensitivity required for some applications, and are often incompatible with immunofluorescent labeling. More recently, bDNA-FISH techniques^6^ have been developed to enhance the sensitivity and specificity of RNA detection, (e.g., PrimeFlow^7^, ViewRNA (Affymetrix) and RNAscope^8^) and permit co-staining by immunofluorescence. bDNA-FISH approaches have also been adapted for imaging of HIV-1 nucleic acids^5, 9^.

**Fig. 1:**
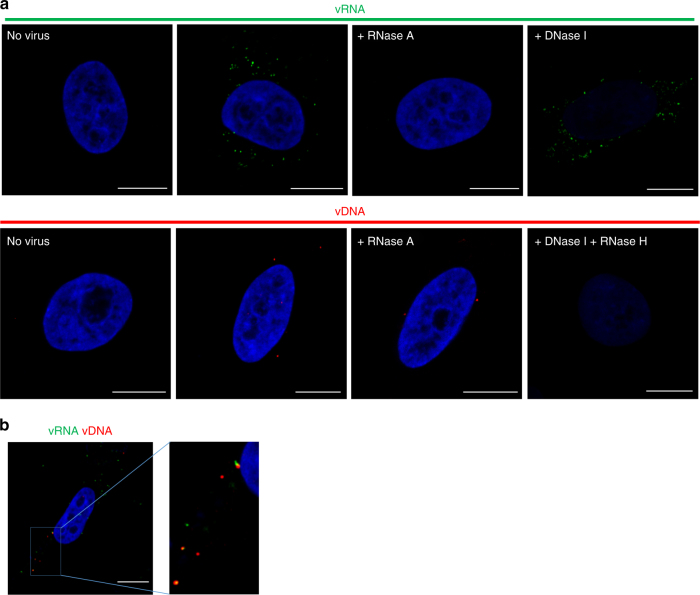
Visualizing HIV nucleic acids. **a** vRNA (top panel): TZM-bl cells were infected with HIV-1 at a multiplicity of infection (MOI) of 1; at 5 hpi cells were fixed and stained. Prior to RNA labeling, cells were treated with buffer alone, RNase A or DNase I. Probe set 1 or PS-1 was used for vRNA (green); nuclei are DAPI stained and shown in blue throughout the figure. vDNA (bottom panel): Prior to DNA labeling cells were treated with RNase A or RNase H followed by DNase I. Probe set PS-3 was used for vDNA (red). **b** Simultaneous detection of vRNA and vDNA of incoming virus in the cytoplasm. TZM-bl cells were infected with HIV-1 at an MOI of 1; at 4 hpi cells were fixed and stained for vRNA (PS-2; green), vDNA (PS-3; red), and nuclei (blue). Enlarged image shows co-localization between vRNA and vDNA. Scale bars represent 10 µm

Based on the RNAscope method^8^, bDNA-FISH protocols that enable visualization of HIV-1 vRNA and vDNA were developed and optimized. Protocols described in Methods section were used with probes that target the *gag* region of HIV-1 RNA, enabling confocal microscopy-based detection of unspliced genomic vRNA in the cytoplasm of cells, shortly after infection with HIV-1 (Fig. 1a, top panel and Supplementary Movie 1). For specific detection of vDNA and not vRNA, probes that target the *gag-pol* region of negative-strand vDNA were used (to avoid labeling of the positive-strand RNA) and conditions were established to optimize denaturation of dsDNA and hybridization of probes (Fig. 1a, lower panels). Labeling of vRNA and vDNA was highly specific, with no fluorescence detected in uninfected cells (Fig. 1a, left panels). This approach permitted specific detection of vRNA or vDNA, as shown by susceptibility or resistance of the fluorescent signal to treatment with RNase A or DNase I (Fig. 1a). In addition, a protocol for simultaneous observation of both HIV vRNA and vDNA was developed. This protocol enabled monitoring of vDNA without very significant reduction of the vRNA signal. Using this method, co-localization of incoming viral genomic vRNA with nascent vDNA was demonstrated at 4 h post-infection (hpi) (Fig. 1b). Hence, this protocol allows single-cell imaging of vRNA and vDNA individually and multiplexed, with sufficient sensitivity to discern what we interpret to be individual virions. Such highly sensitive staining of viral nucleic acid during entry may be used to track the progress of infection.

### Time course of reverse transcription and nuclear entry

**Fig. 2:**
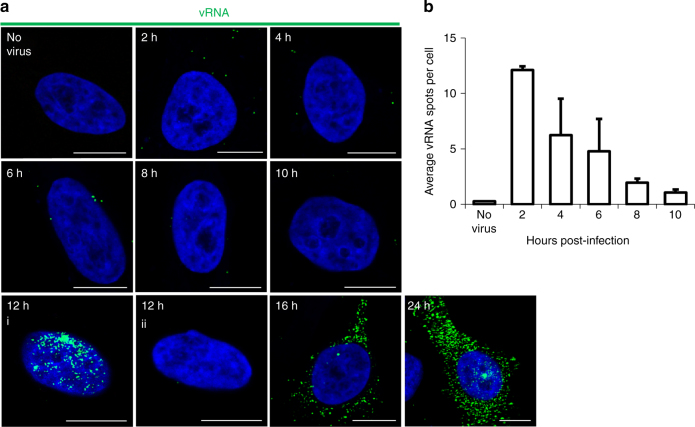
Time course of vRNA detection. **a** TZM-bl cells were infected with HIV-1 at an MOI of 0.2. Cells were then fixed and stained at the indicated times. Cells were stained for vRNA (PS-1; green) and nuclei (blue). At 12 hpi, ~15% of infected cells exhibited a transcription burst (12 h, i) whereas most cells did not yet produce nascent vRNA (12 h, ii). Scale bars represent 10 µm. **b** Foci of vRNA were counted in 300–340 cells per time point, using Gen5 software. The average number vRNA foci per cell was calculated and shown in the graph with standard deviation indicated (*n* = 2 independent experiments)

The protocols for detection of vRNA and vDNA were subsequently used to study the kinetics of HIV infection in a representative cell line (TZM-bl cells). Semi-synchronized cells were infected^10^, fixed, then processed at 2 h intervals for vRNA visualization (Fig. 2). Cells were infected at a relatively low multiplicity of infection (MOI; ~0.2) to prevent saturation of relevant cellular interactions, and foci of vRNA were quantified from multiple randomized images per time point, for more than 300 cells per time point. Under these conditions, automated quantitation produced an estimate of 12 vRNA foci per cell following infection, and 0.26 foci per cell in the absence of infection; the vRNA signal subsequently decreased to an average of 1–2 vRNA foci per cell by 10 hpi (Fig. 2), presumably due to RNase H activity during reverse transcription. In fact, addition of an RNase H inhibitor^11^ counteracted the loss of vRNA signal at 12 hpi (Supplementary Fig. 1). These data suggest that reverse transcription is completed by ~10 hpi, which is consistent with previous estimates based on other experimental techniques that follow uncoating and reverse transcription kinetics^12, 13^. Although not part of these analyzes, the signal may be expected to increase again at later time points, as cells begin transcribing vRNA. At this time, it is no longer possible to track incoming genomes; the density of synthesized vRNA may also presents problems for the enumeration of individual foci. At these later time points, measurement of the fluorescent intensity may more accurately represent the extent of vRNA production.

**Fig. 3:**
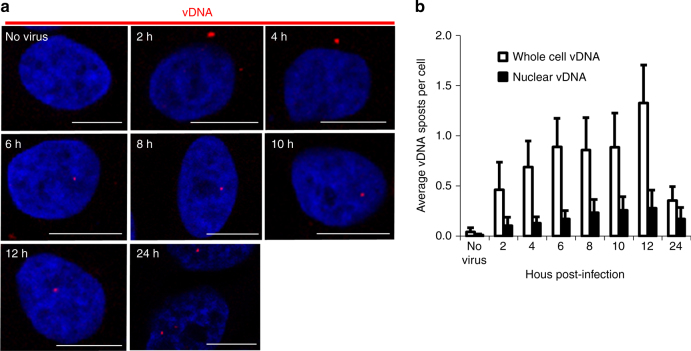
Time course of vDNA detection. **a** TZM-bl cells were infected with HIV-1 at an MOI of ~0.2; at indicated times, cells were fixed and stained for vDNA (PS-3; red) and nuclei (blue). Scale bars represent 10 µm. **b** Foci of vDNA were counted in 300–340 cells per time point, using Gen5 software. The average number of vDNA foci per cell was calculated and shown in the graph with standard deviation indicated (*n* = 3 independent experiments, except for 24 h time point, where *n* = 2)

We additionally monitored the appearance and localization of vDNA, as reverse transcription progressed during HIV-1 infection; HIV-1 vDNA was detected mainly in the cytoplasm of TZM-bl cells, and to a lesser degree in nuclei, at 2 hpi (Fig. 3). Both signals increased over time. Total cellular vDNA reached a peak of 1.3 foci per cell, and nuclear vDNA a peak of 0.27 foci per cell (compared to 0.04 and 0.011 foci per cell in uninfected cells), at 12 hpi, presumably as reverse transcription and nuclear import were completed. The number of vDNA foci was then observed to drop between 12 and 24 h, likely reflecting the degradation of unintegrated vDNA; this effect was more pronounced in the cytoplasm of the cell, presumably because the nuclear foci include integrated proviral vDNA, which is stably maintained. Nuclear localization was determined when foci overlapped the nucleus (DAPI stained), in *x* and *y* dimensions. To verify that the vDNA signal at 2 hpi truly represented newly synthesized reverse transcription products, virions were attached to cells at 4 °C, then stained for vRNA, vDNA and capsid (CA) (Supplementary Fig. 2). It would be expected that only CA and vRNA should be present in HIV-1 particles prior to fusion with a target cell. If a signal was detected for vDNA, that would suggest either that reverse transcription occurred prior to infection, or, more likely, that a significant portion of the vDNA signal is a false positive. Consistent with the accepted model of HIV-1 replication, we observed signals only for the vRNA probe and CA antibody, not for vDNA, confirming that no vDNA is present prior to infection and that our vDNA probe is specific.

In principle, the probes should label both integrated and unintegrated vDNA. To assess labeling in the absence of integration, TZM-bl cells were infected in the presence of 1 µM integrase inhibitor raltegravir (RAL)^14^ (Supplementary Fig. 3). Consistent with its specific inhibition of integration, RAL had no apparent effect on the number of cytoplasmic and nuclear vDNA foci, but nascent vRNA transcription was almost entirely suppressed by the inhibitor. These findings confirm the labeling of integrated and unintegrated vDNA, and suggest that transcription of vRNA coding Gag-Pol is greatly enhanced by integration into the host genome, as previously reported^15^.

### Transcription from integrated vDNA

**Fig. 4:**
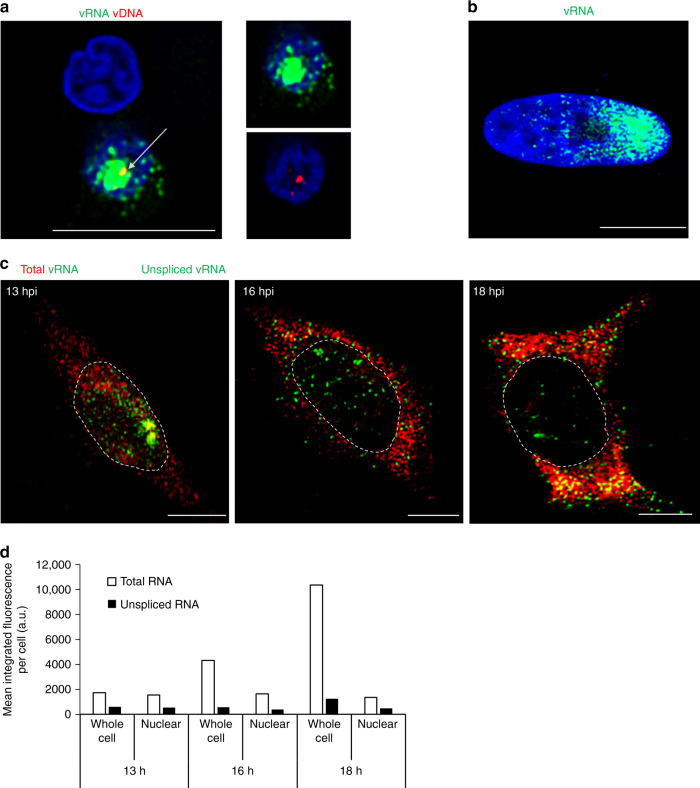
Transcription and splicing of vRNA. **a** Nascent vRNA transcribed from nuclear vDNA in primary cells. Viral transcription site is indicated with a white arrow and appears in yellow because of newly formed vRNA (PS-2; green) and vDNA (PS-3; red) co-localization. Separate red and green channels are shown to the right. PBMCs were infected at an MOI of 0.2; at 48 hpi they were fixed and stained. **b** HIV-1 transcriptional burst phenotype. TZM-bl cells were infected at an MOI of 0.2; at 12 hpi they were fixed and stained with for unspliced vRNA (PS-1; green) and nuclei (blue). **c** Multiplex detection of total and unspliced vRNA informs temporal differences in localization. TZM-bl cells were infected with HIV-1 at an MOI of 0.5. At the indicated times, cells were fixed and stained with PS-4 for total vRNA (spliced and unspliced; PS-4; red), unspliced vRNA (PS-1; green), and nuclei (outline). **d** Spliced and unspliced vRNA were quantified for the whole field of view and within the nuclei. 200–300 cells per time point were analyzed using Gen5 software to measure the total fluorescence signal (the sum of the pixel intensities) per cell, either in the complete field of view (whole cell), or within a nuclear mask (nuclear). Scale bars represent 10 µm

Study of HIV transcription from individual proviruses has been hindered by the lack of an efficient method for simultaneous detection of both vRNA and vDNA in individual cells. By employing this protocol, it is now possible to observe HIV-1 vDNA that is often associated with sites of vRNA transcription (Fig. 4a). Intense accumulation of nascent unspliced vRNA transcripts was observed in ~15% of infected TZM-bl cells shortly after completion of reverse transcription, at 12 hpi, and strictly within the nucleus (Fig. 4b, Supplementary Movie 2); at later time points vRNA distribution matured, with the vRNA transcripts being present predominantly in the cytoplasm (Fig. 2). This phenotype is consistent with reports on transcription of host as well as HIV-1 RNAs occurring in bursts, and accumulation of HIV-1 RNA in the nucleus prior to bursts of vRNA export to the cytoplasm^16–18^.

### RNA splicing and nuclear export

Export of unspliced HIV vRNA is dependent on Rev, a protein coded by a multispliced vRNA^19^. It should therefore be possible to detect spliced vRNA in the cytoplasm before the newly synthesized Rev exports the unspliced vRNA^20^. Conditions were established for simultaneous detection of unspliced and multispliced vRNA in individual cells. Probe set 1 (PS-1) (Supplementary Table 1) targets the *gag* region of the HIV genome and is thus specific for unspliced vRNA. PS-3 targets a region common to spliced and unspliced RNA, and thus detects both; the probes bind distinct regions of the vRNA and there is not significant  overlap in staining, potentially owing to the length of the molecule or the efficiency of hybridization. Comparison of the fluorescent signals at various times post-infection illustrates that multispliced vRNA was present in the cytoplasm hours prior to unspliced vRNA (Fig. 4c, d). At later times, presumably following Rev expression, the majority of vRNA, regardless of splicing, localized to the cytoplasm (Fig. 4c, d). These timelines were consistent with the time course of infection, which used a probe for unspliced vRNA; at 12 hpi the vRNA was exclusively nuclear, but by 16 hpi, vRNA was predominantly cytoplasmic (Fig. 2).

### Multiplex imaging of nucleic acid and protein

**Fig. 5:**
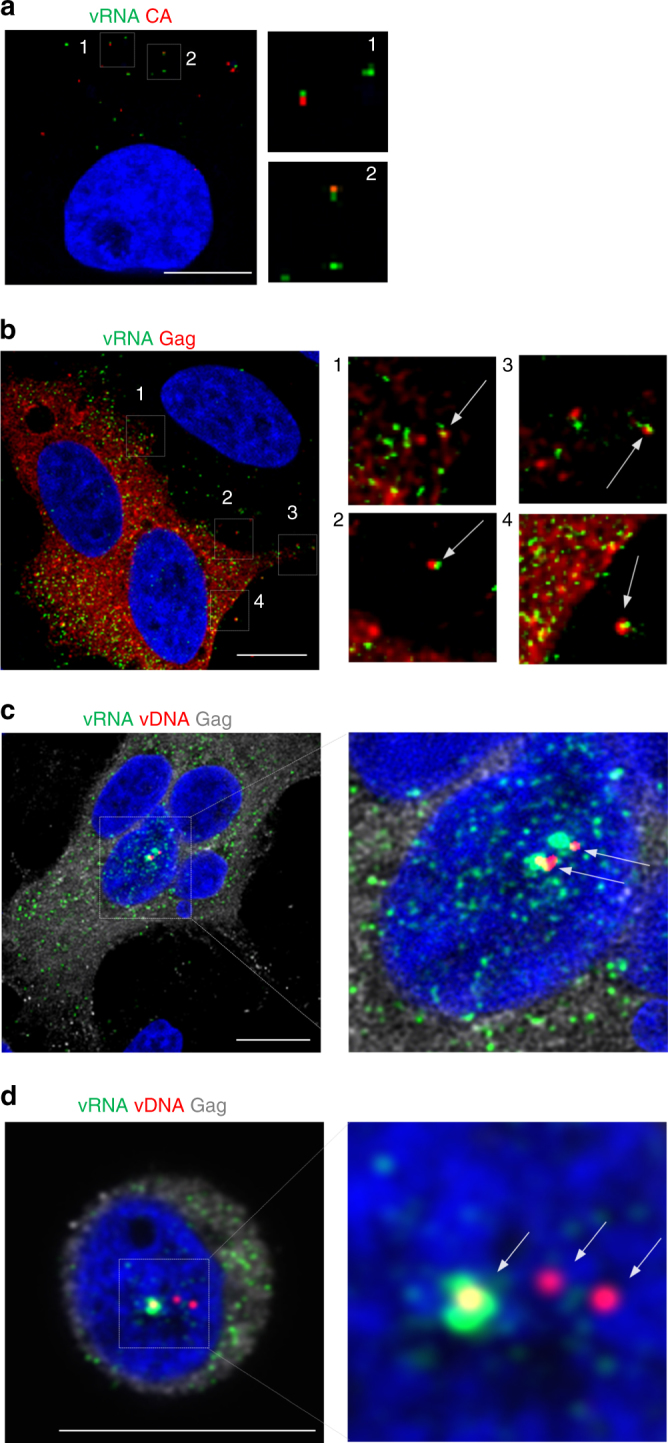
Multiplex visualization of HIV-1 nucleic acids and protein. **a** Examples of colocalized vRNA and capsid (CA). TZM-bl cells were infected with HIV-1 at an MOI of 0.2; at 2 hpi cells were fixed and stained for unspliced vRNA (PS-1; green), CA (red) and nuclei (blue). Enlarged images indicate co-localization between genomic vRNA of incoming virus and CA. **b** Apparent formation of nascent virions. TZM-bl cells were infected with HIV-1 at an MOI of 0.2; at 24 hpi cells were fixed and stained for vRNA (PS-1; green), Gag (red), and nuclei (blue). Arrows indicate putative virions. **c** Multiplex visualization of transcribed vRNA (PS-2; green) from vDNA (PS-3; red, indicated by white arrows) and of translated Gag (gray). TZM-bl cells were infected with HIV-1 at an MOI of 2; at 30 hpi cells were fixed and stained. **d** Discordant expression from nuclear vDNA sites. Jurkat cells were infected with HIV-1 at an MOI of 2; at 24 hpi cells were fixed and stained for vRNA (PS-2; green), vDNA (PS-3; red, indicated with white arrows), Gag (gray), and nuclei (blue). Images were captured with an LSM 880 confocal microscope. Scale bars represent 10 µm

Incompatibility with optimal conditions for immunofluorescence staining is a major shortcoming of conventional FISH methods. In contrast, bDNA-FISH is readily compatible with immunofluorescence^3, 8^, permitting the visualization of vRNA and viral proteins in virions during cell entry (Fig. 5a) and release (Fig. 5b). We combined the protocol for vDNA and vRNA labeling with immunofluorescence, to simultaneously label vDNA, vRNA, and viral proteins. We defined this technique as MICDDRP. MICDDRP allowed co-visualization of transcription from integrated proviruses and translation of viral protein, in this case Gag or CA, at the single-cell level (Fig. 5c, Supplementary Movie 3). Importantly, this development may enable investigation of selective transcription in cells with multiple nuclear vDNA sites, and the factors that affect viral transcription (Fig. 5d, Supplementary Movie 4).

### Imaging with primary cells and virus isolates

To verify the potential utility of this method when handling primary tissue samples, a variety of physiologically relevant human cell types were stained, following infection with NL4–3 (Supplementary Fig. 4) or the primary HIV-1 isolate BaL (Supplementary Fig. 5). These cell types included HEK293T, Jurkat (T lymphocyte cell line), primary peripheral blood mononuclear cells (PBMCs), and monocyte-derived macrophages (MDMs), demonstrating high flexibility for the study of transcription and translation from integrated proviruses in diverse relevant cell types.

**Fig. 6:**
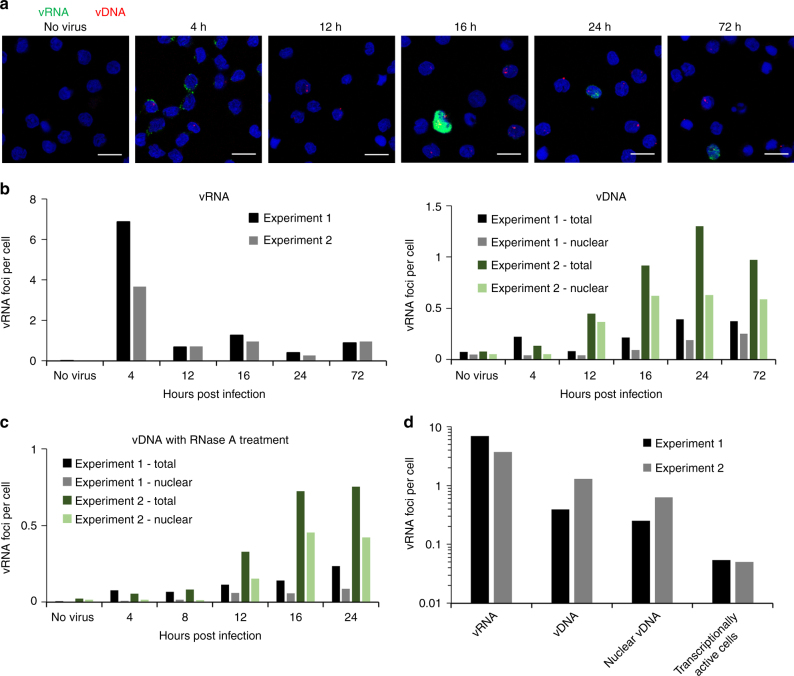
Tracking the early stages of infection of Primary CD4+ T cells. Primary CD4+ T cells were isolated from PBMCs, activated, and infected with HIV-1 NL4–3 at an MOI of 0.05. Cells were then fixed and stained at the indicated times. **a** Cells were stained for vRNA (PS-4; green), vDNA (PS-3; red) and nuclei (blue). Foci of vRNA and vDNA were counted in 1500–3500 cells per time point (4–24 h) or 500–1000 cells (no virus control and 72 h). Foci were counted in the complete field of view (whole cell), or within a nuclear mask (nuclear). **b** Number of vRNA and vDNA foci per cell of two independent experiments were calculated. Number of total vDNA foci or vDNA foci within nuclei (nuclear) are also shown. **c** Staining of vDNA was repeated with RNase A treatment. **d** Peak values for foci of vRNA, vDNA, nuclear vDNA, and transcriptionally active cells were calculated, to allow determination of the efficiency of each stage in the infection process. Scale bars represent 10 µm

A time course comparable to that described in Figs. 2, 3 was also performed using CD4+ T-cells infected with NL4–3, simultaneously stained for vRNA and vDNA (Fig. 6a, b). Similar to the observations with TZM-bl cells, the levels of vRNA were found to be relatively high at early time points, between 4 and 7 foci per cell at 4 hpi, compared to 0.01 foci per cell in uninfected cells. The number of vRNA foci then dropped as reverse transcription proceeded, while vDNA increased over time in measurements both of the whole cell and the nucleus, peaking at 1.3 and 0.6 foci per cell, respectively, following infection, and 0.075 and 0.05 foci per cell in the cell and nucleus, respectively, in the absence of infection. These data demonstrate that the signal to noise ratio is higher for vRNA staining than vDNA (in this low MOI experiment, ~500 to 1 and 10 to 1, respectively), and that vDNA non-specific staining is mainly observed in the nucleus. Treatment of a separate set of samples with RNase A slightly reduced staining in infected and uninfected cells, but did not significantly change the vDNA signal (Fig. 6c). Finally, we compared the peak signals for number of vRNA, vDNA, and nuclear vDNA foci, as well as transcriptionally active cells (Fig. 6d). The signal reduces at each step of the transition from vRNA to actively transcribing cell, as no process is 100% efficient. In this case, we observed a number of vRNA foci per cell that was orders of magnitude greater than the MOI; ~5 vRNA foci per cell yielding an MOI of only 0.05; even at this low MOI, the signal from infection was far higher than the non-specific staining. The conversion rate of cytoplasmic vRNA to an actively infected cell will almost certainly vary depending on cell type used, and potentially between HIV isolates.

### HIV-1 and HIV-2 co-infection

**Fig. 7:**
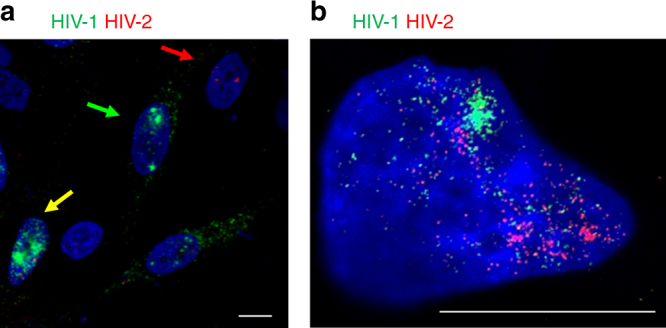
MICDDRP analysis of HIV-1/HIV-2 viral coinfection. TZM-bl cells were infected with HIV-1 and HIV-2 at MOIs of 0.5 and 1, respectively. At 24 hpi cells were fixed and stained for HIV-1 (PS-2; green) and HIV-2 (PS-5; red) vRNA, and nuclei (blue). Images were captured with **a** an Olympus IX81 microscope, or **b** a Leica STED super-resolution system. Scale bars represent 10 µm

More than one million people are dually infected with HIV-1 and HIV-2, and co-infection of cells has been described^21–23^. However, co-infections have been challenging to study owing to significant cross-reactivity of reagents. Here, we combined labeling of vRNA and Gag protein to demonstrate that these reagents selectively label HIV-1 or HIV-2 vRNA in infected cells (Supplementary Fig. 6). These approaches were then applied with either epifluorescent or stimulated emission depletion (STED)^24^ super-resolution microscopy to detect HIV-1/HIV-2 co-infections in cell culture using virus-specific probes. The data demonstrate that it is possible to identify cells infected by HIV-1, by HIV-2, or by both viruses (Fig. 7). In these experiments, 31% of cells were infected with HIV-1, 17% with HIV-2, and 11% were co-infected, a slightly higher figure than would be predicted by random distribution. Future studies will attempt to determine whether this difference reflects interaction between viruses in co-infected cells. These results demonstrate that individual genomes from HIV-1 and HIV-2 can be observed in the same cell, providing the proof of principle for studies of the interplay between the two viruses in co-infected cells.

## Discussion

Here we introduce MICDDRP, a microscopy method that is based on bDNA-FISH^8^, and that can be used to visualize nucleic acid intermediates during HIV replication. This method has been applied to label the viral molecules at various stages of the replication cycle, including the incoming genomic vRNA at early time points, and the various transcribed vRNAs at late time points. Probe sequences have been selected for specific labeling of either the unspliced vRNA that codes for Gag and GagPol and is packaged into particles as the genomic vRNA, or that is transcribed from integrated vDNA and exported in a Rev-dependent manner, or for labeling of total vRNA (spliced and unspliced). Similarly, various vDNA species may be labeled, including the nascent cytoplasmic vDNA (resulting from reverse transcription of the genomic vRNA), integrated proviral vDNA (following integration into the host chromosomes) and non-integrated vDNA (including circular and linear species). Labeling the various species of vRNA and vDNA by this approach is highly sensitive, specific, and compatible with protein immunofluorescence, and can be used to study a wide variety of processes, including reverse transcription of vRNA to vDNA, RNase H activity to degrade vRNA, nuclear import of vDNA, transcription of vRNA from integrated proviral vDNA, splicing of vRNA, export of vRNA from the nucleus, and translation of viral proteins. This approach could also be used for specific questions that we did not address, such as tracking the completion of reverse transcription. Our current vDNA probe labels the first strand of synthesized vDNA, showing that reverse transcription begins in the cytoplasm, consistent with current models of the HIV-1 replication cycle. Labeling of the vDNA requires denaturation of the double-stranded vDNA. If a denaturing protocol were combined with RNase A treatment (to prevent labeling of the positive-stranded vRNA), it should be possible to stain the second (positive) strand of the vDNA, and visualize the site where reverse transcription is completed.

The high sensitivity achieved by bDNA-FISH also lends itself well to diagnostic applications; the high signal to noise ratio results in no “infected” cells being scored in the uninfected controls in our experiments. The RNAscope technology has been applied to identify infected cells in tissue samples, with a sensitivity and specificity comparable to the “gold-standard” method of radiolabeled ISH^9^. The investigators were also able to adapt the technique to label vDNA and vRNA simultaneously in SIV infected tissues, allowing identification of actively and latently infected cells. Beyond identification of latently infected cells, bDNA-FISH has also been combined with immunofluorescent staining to permit highly sensitive identification of reactivated HIV transcription and translation in reactivated, patient-derived samples^25^. Our ongoing studies focus on exploring possible factors that influence the onset of transcription from integrated vDNA. There is particular interest in the characterization of defective proviruses, that is, viruses that are integrated but cannot be transcribed, or can be transcribed but not translated. Latent proviruses accumulate during antiviral therapy, some retain the variable capacity to be reactivated, while others may contribute to pathogenesis despite being incapable of producing infectious virus^26, 27^. The scarcity of latently infected cells renders the problem of latency particularly challenging, and even with the ability to label integrated vDNA, in the absence of transcription, it would require considerable effort to identify and isolate the infected cells. Nevertheless, MICDDRP will be a useful tool, alongside many others, for addressing this major barrier to HIV cure.

In conclusion, we have developed a method for direct visualization of HIV vRNA, vDNA, and protein in the same cell. MICDDRP can be employed to follow early infection events, including the entry of HIV cores in the cytoplasm, the loss of vRNA and appearance of vDNA during reverse transcription, and vDNA trafficking to the nucleus. MICDDRP also allows for differentiation and quantitation of spliced and unspliced RNA. The addition of protein staining allows the expression of viral genes to be addressed, as well as the transcription of RNA from integrated provirus. Ongoing studies focus on exploring possible factors that influence the onset of transcription from integrated vDNA. Similar work has been performed using the EdU labeling method^3^, however that approach requires labeling of the virus during reverse transcription; the bDNA-FISH used in MICDDRP, and similar methods, can be applied to patients-derived samples. This method should be a valuable tool for studies of HIV transcription, latency, co-infection, drug mechanisms of action, and host-cell interactions. Furthermore, MICDDRP should be applicable to a wide range of viruses and other nucleic acid-protein interactions.

## Methods

### Cell culture

TZM-bl (NIH AIDS Reagent Program catalog #8129) are HeLa-derived human epithelial cells that stably express the HIV-1 receptor (CD4) and co-receptors (CXCR4 and CCR5)^28–32^. TZM-bl cells additionally express β-galactosidase and firefly luciferase under the control of the HIV-1 promoter. They were cultured in Dulbecco’s modified Eagle’s medium (DMEM; Gibco), supplemented with 10% fetal bovine serum (FBS) and 2 mM l-glutamine (Gibco), in a humidified incubator at 37 °C with 5% CO_2_. HEK293-FT cells are derived from human embryonic kidney cells, transformed with the SV40 large T antigen (ThermoFisher Scientific; catalog #R70007). They were cultured in the same medium and conditions as described for TZM-bl. Jurkat cells (NIH AIDS Reagent Program catalog #177) are a human, CD4 and CXCR4 positive, T lymphocyte cell line^33^. They were cultured in Roswell Park Memorial Institute 1640 medium (RPMI; Gibco) supplemented with 10% heat-inactivated FBS and 2 mM l-glutamine, in a humidified incubator at 37 °C with 5% CO_2_. THP-1_ATCC_ cells (NIH AIDS Reagent Program catalog #9942) are a monocytic cell line that expresses CD4, CXCR4 and (following activation) CCR5. They were cultured as described for Jurkats. Monocyte-derived macrophages (MDM) and lymphocytes were purified from peripheral blood mononuclear cells (PBMC; STEMCELL Technologies catalog #70025) as previously described^34, 35^. Briefly, MDMs were isolated by adherence to the culture vessel and non-adherent cells were removed and stimulated with 60 U/mL IL-2 (PeproTech) and 4 µg/mL phytohemagglutinin (PrepoTech) for 3–4 days prior to infection. Primary lymphocytes were cultured in RPMI supplemented with 10% heat-inactivated FBS and 60 U/mL IL-2, in a humidified incubator at 37 °C with 5% CO_2_. For time course experiments and clinical isolate infection, primary CD4+ T cells were isolated from PBMCs using EasySep™ Human CD4+ T Cell Enrichment Kit (STEMCELL Technologies), following the manufacturer’s instructions. T cells were cultured and stimulated in RPMI supplemented with 10% heat-inactivated FBS, 2 mM l-glutamine, 60 U/mL IL-2, and 25 µL/mL ImmunoCult Human CD3/CD28/CD2 T-Cell Activator, for 8 days prior to infection. Cells were maintained a humidified incubator at 37 °C with 5% CO_2,_ and every 2–3 days fresh media was added.

### Antibodies and compounds

Capsid antibody, mouse monoclonal anti-p24 (NIH AIDS reagent Program mab-24–2 catalog #6457)^36, 37^, was used at a 1:2000 dilution, and followed by with goat anti-mouse secondary antibodies at a 1:2000 dilution, conjugated to either Alexa Fluor 647 (Invitrogen catalog #A-21235) or Alexa Fluor 568 (Invitrogen catalog #11004). For HIV-1/HIV-2 co-infection experiments, mouse monoclonal anti-p24 (NIH AIDS reagent Program AG3.0 catalog #4121) was used at a 1:250 dilution to stain both HIV-1 and HIV-2 Gag^38^.

RNase H-inhibitor naphthyridinone {4-[(4′-aminomethyl-1,1′-biphenyl)methyl]-1-hydroxy-1,8-naphthyridin-2-one (NAPHRHI)} is derived from 1-hydroxy-1,8-naphthyridin-2(1H)-one. It was prepared as described by Williams et al.^11^. Briefly, the benzyl ester-protected scaffold was saponified, decarboxylated, and the resulting phenol converted to triflate. NAPHRHI was formed by Negishi coupling with a benzylzinc reagent obtained by treating 4-bromobenzyl bromide with diethyl zinc, followed by Suzuki coupling of the bromide with 4-(N-Boc-aminomethyl)phenylboronic acid and by deprotection with HBr^11^ (U.S. patent application 20100056516). Raltegravir^14^ (ISENTRESS/MK-0518) was obtained from the AIDS Reagent Program.

### Nucleic acid probes

All probes were purchased from Advanced Cell Diagnostics. To detect HIV-1 vRNA two anti-sense probes were used; the first probe targets the Gag coding region of HIV-1 (HIV vRNA anti-sense probe-1, PS-1 [probe channel C1 or C2])^39^, and the second probe targets non-Gag-Pol regions coding envelope and accessory proteins (HIV vRNA anti-sense probe-3, PS-3, [probe channel C3]). For HIV-1 vDNA detection, a sense probe targeting the Gag-Pol coding region was used, to avoid binding to the vRNA (HIV vDNA sense probe-2, PS-2, [probes channel ID C1]).

For detection of HIV-2 vRNA, a probe channel C3 anti-sense probe set targeting the *gag* region (HIV-2 vRNA anti-sense probe-5, PS-5, analogous to HIV-1 PS-1), was directly diluted into HIV-1 PS-1 probe at a ratio of 1:50. The mixture was added to coverslips as recommended by the manufacturer. Supplementary Table 1 contains a complete list of the probe sets used in this study.

### HIV-1 infection

Infectious HIV-1 particles were prepared by transfection of 3 × 10^6^ 293FT cells (ThermoFisher Scientific) with 10 µg NL4-3 proviral plasmid^40^ in a 10 cm dish, using 30 µL Fugene 6 (Promega) in 500 µL Opti-MEM (Gibco). Media were changed 16 h post-transfection. Supernatant was collected 48 h after transfection, cleared by centrifugation for 5 min at 700×*g* followed by filtration through a 0.45 µM polyvinylidene fluoride filter, and stored in aliquots at −80 °C. Virus particles were titrated in TZM-bl cells using fivefold serial dilutions in the presence of 20 µg/mL DEAE-Dextran. After 48 h, cells were fixed and the number of blue forming units (bfu) was determined. This titer was used to determine multiplicity of infection (MOI) for future experiments, where MOI of 1 is 1 bfu per cell. It should be noted that differences in experimental conditions (e.g., reduced incubation time, or infecting different cell types) may alter the observed MOI of the experiments, relative to that determined in titration. In these cases, we have quoted the apparent MOI for the experiment, rather than the MOI we would have expected to observe under the titration conditions.

Semi-synchronous infections of TZM-bl cells were performed using 2 × 10^4^ cells plated on 1 mm collagen-coated coverslips (GG-12-Collagen; Neuvitro)^10^. Cells and virus were pre-chilled separately at 4 °C for 10 min, then virus was added to cells and incubated at 4 °C for 20–30 min in the presence of 20 µg/mL DEAE-Dextran before being placed at 37 °C. Virus-containing media were removed after 2 h, and cells were washed twice with Dulbecco’s phosphate buffered saline (DPBS; Sigma). Fresh media were added and the cells were cultured at 37 °C. To harvest, cells were washed twice with DPBS, fixed with 4% paraformaldehyde in DPBS for 30 min at room temperature, and then washed three times with DPBS.

To infect Jurkat and THP-1 cells, 5 × 10^5^ pre-chilled cells were mixed with pre-chilled virus then seeded into 12-well plates with RPMI and 8 µg/mL polybrene. Plates were centrifuged at 1200×*g* for 40 min at 4 °C to promote virus attachment, then placed at 37 °C. Media were removed after 2 h and cells were washed twice by resuspension in DPBS followed by centrifugation at 114×*g* for 5 min. To collect, cells were washed once with DPBS then seeded onto poly-l lysine-coated coverslips (coated overnight with 0.01% w/v poly-L lysine in water (Sigma) at 4 °C, then washed three times with DPBS before use) and allowed to attach for 40 min at room temperature. Cells were fixed with 4% paraformaldehyde in DPBS for 30 min at room temperature, then washed twice with DPBS. Infections of primary cells were performed as described for Jurkat and THP-1 cells.

For the time course of infection in primary CD4+ T cells, HIV-1 NL4-3 was used, concentrated with Lentivirus precipitation solution (Alstem), following the manufacturer’s instructions. For the infection of primary CD4+ T cells, the HIV-1_Ba-L_ isolate was used^41, 42^, as provided by the AIDS Reagent program. For both experiments, 3–5 × 10^5^ pre-chilled CD4+ T cells (activated 8 days earlier) were mixed with pre-chilled virus, then seeded into 12-well plates with RPMI and 8 µg/mL polybrene. Plates were centrifuged at 1200×*g* for 1 h at 16–20 °C, then placed at 37 °C. Media were removed after 4 h and cells were washed twice by resuspension in DPBS followed by centrifugation at 300×*g* for 5 min. To collect, cells were washed once with DPBS then seeded onto poly-l-lysine-coated coverslips (coated overnight with 0.01% w/v poly-l-lysine (Sigma) in water at 4 °C, then washed three times with DPBS before use) and allowed to attach for 10 min at 37 °C. Cells were fixed with 4% paraformaldehyde in DPBS for 30 min at room temperature, then washed twice with DPBS.

### HIV-1 and HIV-2 coinfections

HIV-2 infectious particles were produced by transfection of 3 × 10^6^ 293FT cells with 10 µg HIV-2.ST proviral plasmid^43^ in a 10 cm dish, using 30 µL Fugene 6 in Opti-MEM. HIV-1 and HIV-2 particles were added simultaneously to TZM-bl cells, at MOIs of 0.5 and 1, respectively, then incubated for 24 h at 37 °C. Cells were then collected for staining as described for HIV-1 infections above.

### In situ vRNA detection

HIV-1 vRNA in cells was probed using RNAscope reagents (Advanced Cell Diagnostics). The manufacturer’s protocol was used with some modifications^8^. Following fixation, cells were dehydrated by removal of DPBS and sequential replacement with 50%, 70% then 100% ethanol, incubating the samples for 5 min at room temperature in each solution. Ethanol solutions were prepared as volume-to-volume ratios in ultrapure water (Synergy; Millipore). The 100% ethanol was replaced with fresh 100% ethanol and incubated at room temperature for a final 10 min. At this point coverslips can be stored in 100% ethanol at −20 °C. To rehydrate cells, the sequence was reversed and the cells were incubated for 2 min at room temperature in each solution; the cells were not allowed to dry in air at any time during the process. Finally, 50% ethanol was replaced with PBS and the cells were hydrated at room temperature for 10 min. Cells were then washed with 0.1% Tween in PBS for 10 min, and twice more in PBS for 1 min. Prior to hybridization, coverslips were immobilized on glass slides; a small drop of nail polish was placed on a glass slide and the coverslip edge was placed on the nail polish drop. Using an ImmEdge hydrophobic barrier pen (Vector Laboratories), a circle was drawn around the coverslip and PBS was added to prevent sample dehydration. The manufacturer’s protease solution (Pretreat 3) was diluted in PBS as appropriate prior to the experiment, (a 1:2 dilution was used when staining incoming virions and a 1:15 dilution when staining for transcription and translation), and incubated in a humidified HybEZ oven (Advanced Cell Diagnostics) at 40 °C for 15 min. Protease solution was discarded and the slides were washed twice by immersion in PBS at room temperature for 1 min. Specific pre-designed anti-sense probes (Supplementary Table 1) that recognize the HIV-1 vRNA were added to the coverslip, as specified by the manufacturer (Advanced Cell Diagnostics): C1 probes were added directly, C3 probes (PS-3 and PS-5), were diluted 1:50 in probe dilution buffer or in C1 probe. Probes were allowed to hybridize with the samples in a humidified HybEZ oven at 40 °C for 2 h. The probes were then discarded and the coverslips washed twice using the proprietary wash buffer. All wash steps were performed on a rocking platform at room temperature for 2 min, using the proprietary wash buffer. The probes were visualized by hybridizing with preamplifiers, amplifiers, and finally, fluorescent label. Pre-amplifier 1 (Amp 1-FL) was hybridized to its cognate probe in a humidified HybEZ oven at 40 °C for 30 min. Samples were washed twice, then hybridized with Amp 2-FL in a humidified HybEZ oven at 40 °C for 15 min, to suppress background staining. After a further two washes, amplifier (Amp 3-FL) was hybridized to Amp 1-FL in a humidified HybEZ oven at 40 °C for 30 min. Samples were washed twice, then fluorescent label Amp 4-FL was hybridized to Amp 3-FL in a humidified HybEZ oven at 40 °C for 15 min, then washed twice more. For HIV-1 experiments, the labeled probe set Amp 4A-FL was used, labeling the C1 probes with Alexa 488, and the C3 probe (PS-4) with Atto 647. For HIV-1/HIV-2 co-staining experiments, the Amp 4C-FL was used, labeling the C1 probe with Atto 550 and the C3 probe (PS-5) with Alexa 488.

For subsequent protein staining, the RNA-labeled samples were washed with PBS and immunostaining was performed as described below. Otherwise, the final step was to counter-stain nuclei with the manufacturer-supplied 4′,6′-diamino-2-phenylindole (DAPI, Advanced Cell Diagnostics) for 30 s at room temperature, then remove the DAPI, wash twice with PBS and immediately mount the coverslips on slides using Prolong Gold Antifade (Invitrogen).

### In situ vDNA detection

For vDNA detection, following protease treatment, samples were washed three times with ultrapure water for 2 min, treated with 5 mg/mL RNase A (Qiagen) in PBS for 30 min at 37 °C, washed three times for 2 min with ultrapure water and heated 50 °C for 30 min with hybridization buffer (1.7 M ethylene carbonate, 100 µg/mL dextran sulfate [average MW >500 kDa], 600 mM NaCl, 0.1% Tween-20 and 10 mM sodium citrate, pH 6.2) to denature the double-stranded DNA^44^. For labeling of vDNA in PBMCs, denaturation was performed at 67 °C for 10 min^45^. The hybridization buffer was then removed and the probe (in the manufacturer-supplied buffer) was diluted 1:1 with hybridization buffer, then allowed to hybridize with the samples in a humidified HybEZ oven at 40 °C for 2 h. Washes and hybridization of pre-amplifiers and amplifiers to the probes were performed as described for vRNA.

### Simultaneous in situ vRNA and vDNA detection

For co-staining of vRNA and vDNA, samples were first treated as described for vRNA staining, up to the point of vRNA probe hybridization for 2 h at 40 °C. Following vRNA probe hybridization, samples were washed twice in the manufacturer’s wash buffer, then incubated with vDNA probes for 2 h at 40 °C in hybridization buffer, but without heating, to avoid loss of the vRNA signal. The hybridization of pre-amplifiers and amplifiers to the probes was performed as described for vRNA. Addition of the vRNA probe prior to denaturation of the DNA prevents hybridization of the probe to the coding strand of the vDNA.

### Immunostaining

Staining for protein was performed after staining for nucleic acids. Coverslips were blocked with 1% bovine serum albumen (BSA) and 10% FBS in PBS containing 0.1% Tween-20 (PBST) at room temperature for 1 h. Capsid protein (CA), or the capsid domain of Gag protein, was then probed with anti-p24 diluted 1:2000 in PBST supplemented with 1% BSA and incubated at room temperature for 1 h. Samples were washed twice in PBST at room temperature for 10 min with rocking. Fluorescently labeled secondary antibodies were used at 1:2000 and incubated at room temperature for 1 h, then the samples washed once with in PBST with at room temperature for 10 min with rocking. Nuclei were stained either using the DAPI provided in the RNAscope kit, or with 0.5 µg/mL Hoescht-33258 in PBS at room temperature for 10 min. Coverslips were washed twice in PBST at room temperature for 10 min with rocking and washed once with PBS for 1 min. Finally, coverslips were mounted on slides using Prolong Gold Antifade, then sealed with nail polish.

### Imaging and imaging quantification

Unless otherwise stated, images were taken with a Leica TCS SP8 inverted confocal microscope equipped with a ×63/1.4 oil-immersion objective, and a tunable supercontinuum white light laser. The excitation/emission bandpass wavelengths used to detect DAPI, Alexa 488, ATTO 550, Alexa 568, and Alexa 647 were set to 405/420–480, 488/505–550, 550/560–610, 568/580–630, and 647/655–705 nm, respectively. Confocal data sets were deconvolved using the Huygens Professional software (Version 16.10, SVI). Some images were captured using a Zeiss LSM 880 Airyscan confocal microscope. When images were captured with the LSM 880, a Plan-Apochromat ×63/1.4 oil objective was used in the Airyscan super-resolution mode. Following full 3D capture with a Z-step size of 0.16 microns, raw images were subjected to the standardized Airyscan processing routine (which includes photon reassignment and linear parametric 3D deconvolution). Movies were produced based on volume-rendered Z-stacks of confocal images using LAS X (Leica Microsystems) or ZEN (Zeiss) imaging software, and edited using Fiji^46, 47^. For time course experiments, fields of view were selected randomly based on the DAPI signal. Images were taken of the DAPI, vRNA, and vDNA staining (10 images per sample using the ×63 objective). The numbers of nuclei and vRNA or vDNA foci were quantified using Gen5 software (BioTek), and the number of foci for each image was divided by the number of nuclei in the image to determine the average number of foci per cell. To determine the number of nuclear vDNA foci, a mask was applied the image based on the DAPI stain; vDNA foci that fell within the DAPI mask were counted as nuclear vDNA foci.

HIV-1/HIV-2 coinfection images were acquired with an Olympus IX81 microscope, using a ×40 objective. Super-resolution images of HIV-1/HIV-2 co-infected cells were acquired with a Leica TCS SP8 STED inverted confocal microscope, using a ×100/1.4 objective, the excitation/emission bandpass wavelengths/depletion laser used to detect DAPI, Alexa 488, and ATTO 550 were set to 405/420–480, 488/495–550/592, and 550/560–610/660 nm, respectively.

### Data availability

All data supporting the findings in this paper are included in the main text and Supplementary Information. All relevant data are available from the authors upon request.

## Electronic supplementary material

Supplementary Information

Descriptions of Additional Supplementary Files

Supplementary Movie 1

Supplementary Movie 2

Supplementary Movie 3

Supplementary Movie 4
