# Supplementary material for: Multiplex single-cell visualization of nucleic acids and protein during HIV infection
Source: Nat Commun. 2017 Dec 1;8:1882. doi: 10.1038/s41467-017-01693-z (PMC5709414; doi:10.1038/s41467-017-01693-z)
Supplement: Supplementary file 1 — Supplementary Information [file 41467_2017_1693_MOESM1_ESM.pdf]

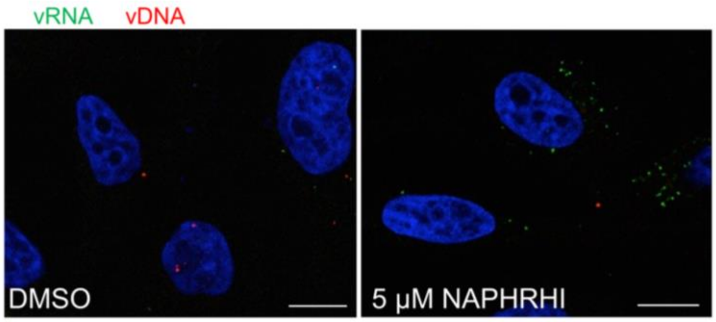

Supplementary Figure 1. Degradation of vRNA during reverse transcription. TZM-bl cells were treated with DMSO (control) or NAPHRHI (RNase H inhibitor) at 5 µM for 1 h, then infected with HIV-1 at an MOI of 0.4; at 12 hpi, cells were fixed and stained for HIV-1 vRNA (PS-2; green), vDNA (PS-3; red), and nuclei (blue). Scale bars represent 10 µm.

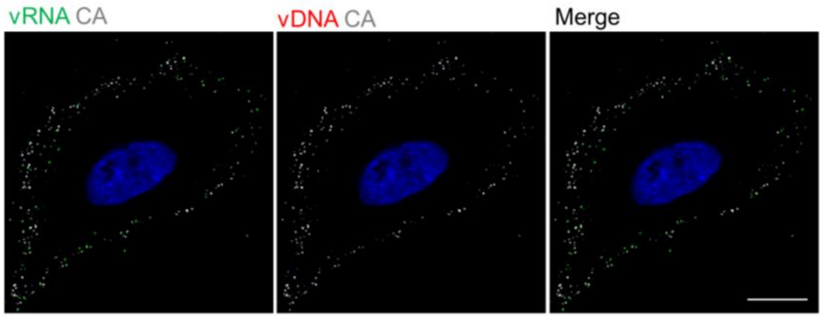

Supplementary Figure 2. Staining for vRNA and vDNA in particles at the plasma membrane. TZM-bl cells were spinoculated with HIV-1 particles at an MOI of 2, for 2 h at 4 ºC, to prevent membrane fusion. The samples were then fixed immediately and stained for vRNA (PS-1; green), vDNA (PS-3; red), and CA (white). Scale bar represents 10 µm.

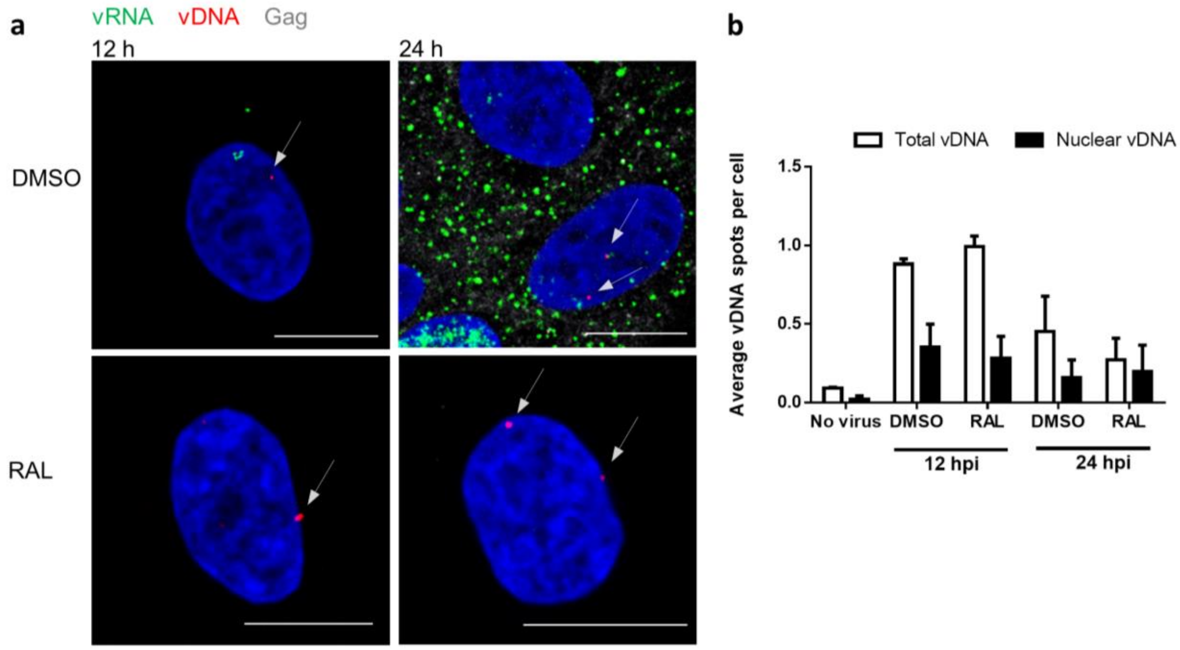

Supplementary Figure 3. Inhibition of HIV-1 integration. **(a)** TZM-bl cells were infected with HIV-1 at an MOI of ~0.3, in the presence of 0.5% DMSO or 1 µM raltegravir (RAL)/0.5% DMSO. Cells were fixed at the indicated times and stained for vRNA (PS-2; green), vDNA (PS-3; red, indicated by white arrows), Gag (gray), and nuclei (blue). Scale bars represent 10 µm. **(b)** Foci of vDNA were quantified for 10 fields of view for each condition, using Gen5 software. Average results are plotted in the graph with standard deviation indicated (n = 2 or 3 independent experiments).

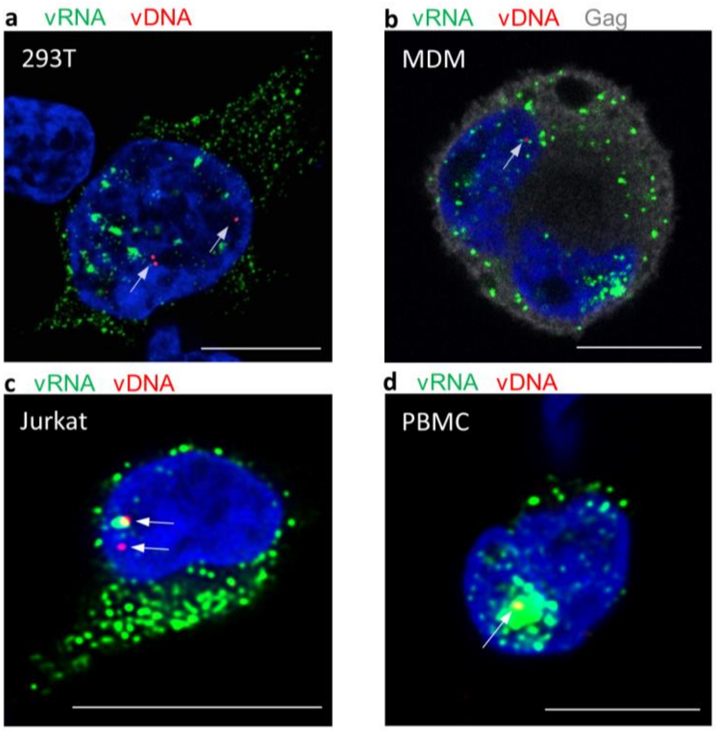

Supplementary Figure 4. Multiplex vRNA and vDNA labeling in diverse cell lines. **(a)** HEK293T cells were infected with VSV-G-pseudotyped HIV-1 at an MOI of ~2; at 15 hpi cells were fixed and stained for vRNA (PS-2; green), vDNA (PS-3; red), and nuclei (blue). **(b)** Monocyte derived macrophages were infected with HIV-1 at an MOI of ~2; at 48 hpi cells were fixed and stained for vRNA (PS-2; green), vDNA (PS-3; red), Gag (gray), and nuclei (blue). **(c)** Jurkat cells were infected with HIV-1 at an MOI of 2; at 24 hpi cells were fixed and stained as in (a). **(d)** Primary lymphocytes were infected with HIV-1 at an MOI of 0.2; at 24 hpi cells were fixed and stained as in (a). Scale bars represent 10 µm.

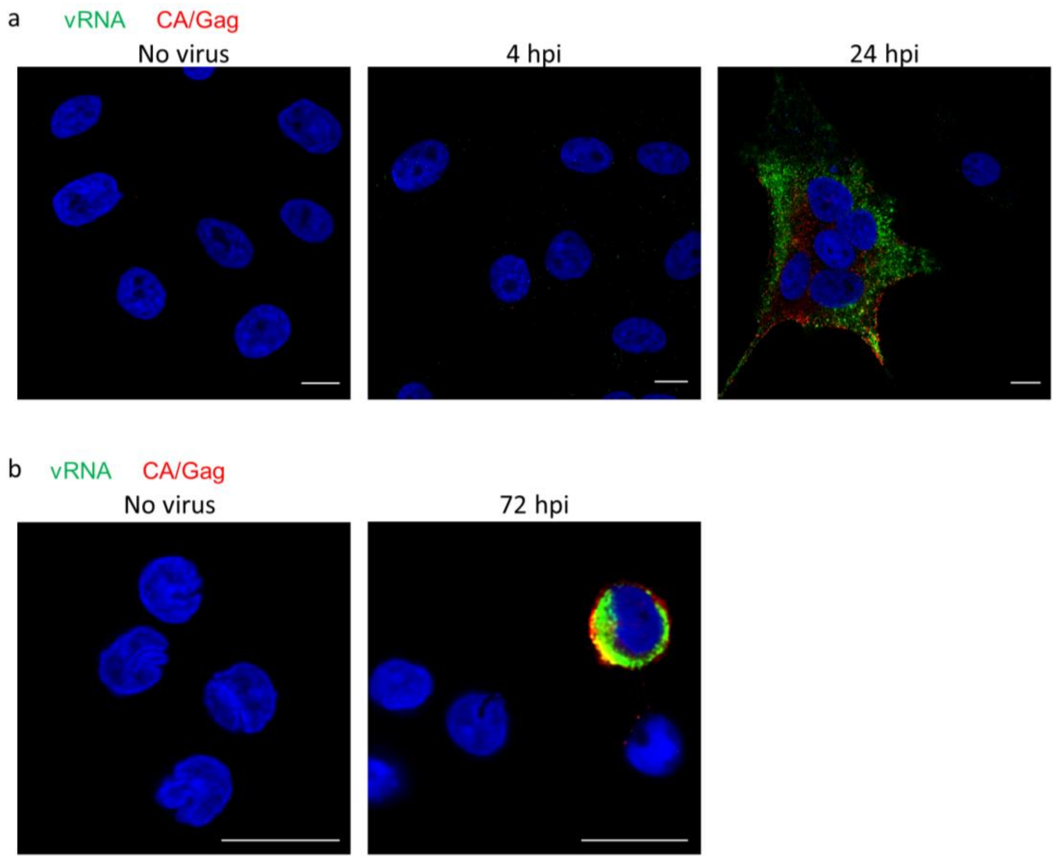

Supplementary Figure 5. vRNA and Capsid/Gag labeling of HIV-1 clinical isolate. **(a)** TZM-bl cells were infected with HIV-$1_{Ba\text{-}L}$ clinical isolate at an MOI of ~1; at 4 hpi and 24 hpi cells were fixed and stained for vRNA (PS-1; green), CA/Gag (red), and nuclei (blue). **(b)** Primary CD4+ T cells were infected with HIV-$1_{Ba\text{-}L}$ clinical isolate at an MOI of ~0.02; at 72 hpi cells were fixed and stained for vRNA (PS-1; green), Gag (red), and nuclei (blue). Scale bars represent 10 µm.

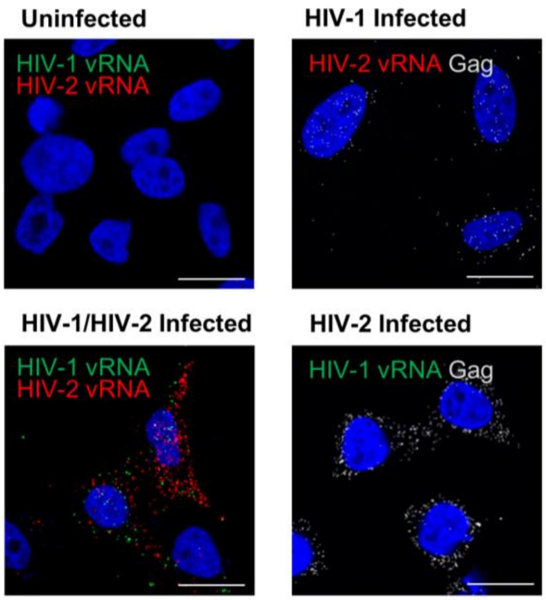

Supplementary Figure 6. Specificity of HIV-1 and HIV-2 labeling. TZM-bl cells were infected with HIV-1 and HIV-2 at MOIs of 0.5 and 1, respectively. At 24 hpi cells were fixed and stained for HIV-1 (PS-2; green) or HIV-2 (PS-5; red) vRNA, Gag (grey) and nuclei (blue). No vRNA foci can be observed in productively infected cells (Gag is being produced) that are labeled with non-cognate vRNA labels (top right and bottom right images). Images were captured with an Olympus IX81 microscope. Scale bars represent 10 µm.

Supplementary Table 1

**Multiplex Immunofluorescent Cell-based Detection Probes Sets**				
**Probe Set (PS)**	Name	ACD Catalog No.	Number of ZZ pairs	Description
**PS-1**	HIV-1 vRNA anti-sense probe-set 1	311921-C1	10	Anti-sense probe targeting within 801-1393 bp of HIV-1 (gag). Accession No NC_001802.1
**PS-2**	HIV-1 vRNA anti-sense probe-set 1	446211-C2	10	Anti-sense probe targeting within 801-1393 bp of HIV-1 (gag). Accession No NC_001802.1
**PS-3**	HIV-1 vDNA sense probe-set 2	317701-C1	60	Sense probe targeting within 507-4601 bp of HIV-1 (gag, pol) Accession No NC_001802.1
**PS-4**	HIV-1 vRNA anti-sense probe-set 3	317711-C3	40	Anti-sense probe targeting within 4988-9181 bp of HIV-1. (vif, vpr, tat, rev, vpu, env, and nef) Accession No NC_001802.1
**PS-5**	HIV-2 vRNA anti-sense probe-set 5	446221-C3	20	Anti-sense probe targeting within 1379-2447 bp of HIV-2 (gag). Accession No L07625.1
