# Supplementary material for: Multiplex single-cell visualization of nucleic acids and protein during HIV infection
Source: Nat Commun. 2017 Dec 1;8:1882. doi: 10.1038/s41467-017-01693-z (PMC5709414; doi:10.1038/s41467-017-01693-z)
Supplement: Supplementary file 2 — Descriptions of Additional Supplementary Files [file 41467_2017_1693_MOESM2_ESM.docx]

**Descriptions of Additional Supplementary Files**

File Names: Supplementary Movie 1

Descriptions: Incoming HIV-1 vRNA. TZM-bl cells were infected with HIV-1 at an MOI of 5. At 5 hpi cells were fixed and probed for vRNA (PS-1; green), and nuclei (blue). Z-stacks were captured with a Leica SP8 confocal microscope using a 63x/1.4 oil-immersion objective. Scale bar represents 10 µm.

File Names: Supplementary Movie 2

Descriptions: Burst transcription of HIV-1 vRNA. TZM-bl cells were infected with HIV-1 at an MOI of 1. At 12 hpi cells were fixed and probed for vRNA (PS-1; green), and nuclei (blue). Z-stacks were captured with a Leica SP8 confocal microscope using a 63x/1.4 oil-immersion objective. Scale bar represents 10 µm.

File Names: Supplementary Movie 3

Descriptions: HIV-1 vRNA, vDNA and Gag detection in Jurkat cells. Jurkat cells were infected with HIV-1 at an MOI of 2. At 24 hpi cells were fixed and probed for vRNA (PS-2; green), vDNA (PS-3; red), Gag (gray), and nuclei (blue). Z-stacks were captured with a Zeiss LSM 880 confocal microscope using a 63x/1.4 oil-immersion objective. Scale bar represents 5 µm.

File Names: Supplementary Movie 4

Descriptions: Jurkat cells were infected with HIV-1 at an MOI of 2. At 24 hpi cells were fixed and probed for vRNA (PS-2; green), vDNA (PS-3; red), Gag (gray), and nuclei (blue). Z-stacks were captured with a Zeiss LSM 880 confocal microscope using a 63x/1.4 oil-immersion objective. Scale bar represents 5 µm.
